# Blue light reduces photosynthetic efficiency of cyanobacteria through an imbalance between photosystems I and II

**DOI:** 10.1007/s11120-018-0561-5

**Published:** 2018-07-19

**Authors:** Veerle M. Luimstra, J. Merijn Schuurmans, Antonie M. Verschoor, Klaas J. Hellingwerf, Jef Huisman, Hans C. P. Matthijs

**Affiliations:** 1 Department of Freshwater and Marine Ecology, Institute for Biodiversity and Ecosystem Dynamics, University of Amsterdam, PO Box 94248, 1090 GE Amsterdam, The Netherlands; 2 Wetsus, European Centre of Excellence for Sustainable Water Technology, Oostergoweg 9, 8911 MA Leeuwarden, The Netherlands; 3 Swammerdam Institute for Life Sciences, University of Amsterdam, PO Box 94248, 1090 GE Amsterdam, The Netherlands; 4 Present Address: KWR Watercycle Research Institute, PO Box 1072, 3430 BB Nieuwegein, The Netherlands

**Keywords:** Blue light, Cyanobacteria, Photosynthesis, Photosystems, Phycobilisomes, *Synechocystis* PCC 6803

## Abstract

Several studies have described that cyanobacteria use blue light less efficiently for photosynthesis than most eukaryotic phototrophs, but comprehensive studies of this phenomenon are lacking. Here, we study the effect of blue (450 nm), orange (625 nm), and red (660 nm) light on growth of the model cyanobacterium *Synechocystis* sp. PCC 6803, the green alga *Chlorella sorokiniana* and other cyanobacteria containing phycocyanin or phycoerythrin. Our results demonstrate that specific growth rates of the cyanobacteria were similar in orange and red light, but much lower in blue light. Conversely, specific growth rates of the green alga *C. sorokiniana* were similar in blue and red light, but lower in orange light. Oxygen production rates of *Synechocystis* sp. PCC 6803 were five-fold lower in blue than in orange and red light at low light intensities but approached the same saturation level in all three colors at high light intensities. Measurements of 77 K fluorescence emission demonstrated a lower ratio of photosystem I to photosystem II (PSI:PSII ratio) and relatively more phycobilisomes associated with PSII (state 1) in blue light than in orange and red light. These results support the hypothesis that blue light, which is not absorbed by phycobilisomes, creates an imbalance between the two photosystems of cyanobacteria with an energy excess at PSI and a deficiency at the PSII-side of the photosynthetic electron transfer chain. Our results help to explain why phycobilisome-containing cyanobacteria use blue light less efficiently than species with chlorophyll-based light-harvesting antennae such as *Prochlorococcus*, green algae and terrestrial plants.

**Electronic supplementary material:**

The online version of this article (10.1007/s11120-018-0561-5) contains supplementary material, which is available to authorized users.

## Introduction

Almost 140 years ago, professor Theodor Engelmann showed that light color plays an important role in photosynthesis (Engelmann 1882). In his classic experiment, Engelmann placed a filamentous green alga from the genus *Cladophora* on a microscopic slide, which he illuminated through a prism glass, thus dividing sunlight into separate wavelengths across the filament. By introducing aerotactic bacteria and observing in which regions of visible light these bacteria aggregated, he established that photosynthetic oxygen (O_2_) production occurred in red and blue light, thereby creating the first “living” action spectrum of chlorophyll.

In the following years, Engelmann continued his studies with cyanobacteria from the genus *Oscillatoria*, demonstrating that in these cyanobacteria, not only red and blue light but also orange light resulted in high O_2_ production rates (Engelmann 1883, 1884). Engelmann’s findings were criticized for many years, but 60 years later, his results were confirmed by Emerson and Lewis, who showed that the phycobiliproteins of cyanobacteria and red algae play a key role in light-harvesting for photosynthesis (Emerson and Lewis 1942). We now know that these phycobiliproteins make up specialized light-harvesting antennae, called phycobilisomes (PBSs), consisting of an allophycocyanin core and stacked rods of phycocyanin often in combination with phycoerythrin. These phycobiliproteins consist of an apo-protein and one or more chromophores, also known as bilins, including phycocyanobilin absorbing orange light (620 nm), phycoerythrobilin absorbing green light (545 nm), and phycourobilin absorbing blue-green light (495 nm) (Grossman et al. 1993; Tandeau de Marsac 2003; Six et al. 2007). Recent reviews on the structure and function of PBSs are provided by Tamary et al. (2012), Watanabe and Ikeuchi (2013), and Stadnichuk and Tropin (2017).

Light energy absorbed by PBSs is effectively transferred via allophycocyanin to the chlorophyll *a* (Chl *a*) pigments in the photosystems (Arnold and Oppenheimer 1950; Duysens 1951; Lemasson et al. 1973). It has long been assumed that most PBSs transfer their energy to photosystem II (PSII). It is now well established, however, that cyanobacteria are able to re-balance excitation energy by moving PBSs between photosystem I (PSI) and PSII in a process called state transitions (van Thor et al. 1998; Mullineaux 2008). As a consequence of these state transitions, which occur at time scales of seconds to minutes, the PBSs associate with PSII (state 1) or PSI (state 2) and transfer the absorbed light energy to the reaction center of the photosystem they are associated with (Kirilovsky 2015). At longer time scales, cyanobacteria can also adjust their PSI:PSII ratio to optimize their photosynthetic activity under different environmental conditions (Fujita 1997). In cyanobacteria, the PSI:PSII ratio generally ranges between 5:1 and 2:1 depending on light quality and intensity, which is higher than the approximately 1:1 ratio often found in eukaryotic phototrophs (Shen et al. 1993; Murakami et al. 1997; Singh et al. 2009; Allahverdiyeva et al. 2014; Kirilovsky 2015).

Since blue and red light are both strongly absorbed by Chl *a*, and the intermediate wavelengths by the different phycobiliproteins, one would expect that these light colors are all used for photochemistry at approximately equal efficiency. However, several studies have described that blue light yields lower O_2_ production rates than red light in cyanobacteria (Lemasson et al. 1973; Pulich and van Baalen 1974; Jørgensen et al. 1987; Tyystjärvi et al. 2002), in cyanolichens (Solhaug et al. 2014), and also in PBS-containing red algae (Ley and Butler 1980; Figueroa et al. 1995). Furthermore, other studies noted that blue light resulted in lower growth rates in a variety of cyanobacteria (Wyman and Fay 1986), including *Synechocystis* sp. PCC 6803 (Wilde et al. 1997; Singh et al. 2009; Bland and Angenent 2016), *Synechococcus* sp. (Choi et al. 2013), and *Spirulina platensis* (Wang et al. 2007; Chen et al. 2010).

A possible explanation for their poor performance in blue light might be that most chlorophyll of cyanobacteria is located in PSI (Myers et al. 1980; Fujita 1997; Solhaug et al. 2014; Kirilovsky 2015), and hence, blue light induces high PSI but low PSII activity. This phenomenon is also known from fluorescence studies, where the use of blue measuring light complicates interpretation of the fluorescence signal of cyanobacteria (Campbell et al. 1998; Ogawa et al. 2017). However, although several of the above-cited studies measured growth rates and/or pigment composition in different light colors, they did not report on, e.g., O_2_ production, PSI:PSII ratios, or state transitions. Conversely, other studies measured O_2_ production rates or PSI:PSII ratios but did not measure growth rates or other relevant parameters. To our knowledge, more comprehensive studies of the photophysiological response of cyanobacteria to blue light are largely lacking, and no clear consensus has yet been reached on the question why their photosynthetic activity might be hampered by blue light.

In this study, we compare the effect of blue light with that of orange and red light on the model cyanobacterium *Synechocystis* sp. PCC 6803. This cyanobacterium uses Chl *a* in its photosystems and phycocyanin but not phycoerythrin in its phycobilisomes, and hence effectively absorbs blue, orange, and red light. We investigate its photosynthetic performance, the composition of its photosynthetic machinery, and its growth rate at different light intensities for all three colors, and compare these results with growth of the green alga *Chlorella sorokiniana* and of other cyanobacteria containing phycocyanin and phycoerythrin. The experiments will make use of blue, orange, and red LED light with narrow-band wavelengths, which allows more precise investigation of the photosynthetic response to different light colors than the broad-band light filters used in the older literature. Our results demonstrate that blue light has a major impact on the photophysiology of cyanobacteria.

## Materials and methods

### Strains and culture conditions

The freshwater cyanobacterium *Synechocystis* sp. strain PCC 6803 and green alga *Chlorella sorokiniana* 211-8K were pre-grown in light-limited continuous cultures (1.8 L) as described by Huisman et al. (2002). The continuous cultures were provided with BG-11 medium supplemented with 5 mM Na_2_CO_3_ and maintained at 30 °C at a dilution rate of *D* = 0.015 h^−1^ (0.36 day^−1^). Steady-state culture densities were 7.8 × 10^7^ cells mL^−1^ for *Synechocystis* and 2.4 × 10^7^ cells mL^−1^ for *C. sorokiniana*. The cultures were mixed by bubbling with CO_2_-enriched air (2% v/v) flowing at a rate of 30 L h^−1^. Lighting was provided by white fluorescent tubes (Philips Master TL-D 90 De Luxe 18 W/965, Philips Lighting B.V., Eindhoven, The Netherlands) at an incident light intensity of 35 µmol photons m^−2^ s^−1^. Light intensities (PAR) were measured with an LI-250 light meter (LI-COR Biosciences, Lincoln, NE, USA). The CO_2_ concentration of the gas mixture was regularly monitored using an Environmental Gas Monitor for CO_2_ (EGM-4; PP Systems, Amesbury, MA, USA).

The marine cyanobacteria *Synechococcus* sp. strain CCY 9201 (formerly known as BS4) and *Synechococcus* sp. strain CCY 9202 (formerly known as BS5) were pre-grown in Erlenmeyer flasks at 21 °C with incident white light of 15 µmol photons m^−2^ s^−1^. Both *Synechococcus* strains were grown in brackish medium as described by Stomp et al. (2004).

For batch experiments using *Synechocystis* and *C. sorokiniana*, samples from each starter culture were diluted in fresh BG-11 medium supplemented with 20 mM NaHCO_3_. For batch experiments using *Synechococcus* sp. CCY 9201 and CCY 9202, samples from each pre-culture were diluted in fresh brackish medium. Each culture was diluted to an OD_750_ of 0.04 to allow light acclimation already in early cell generations, before density increases could be detected.

### Batch experiments using an incubation shaker

**Fig. 1:**
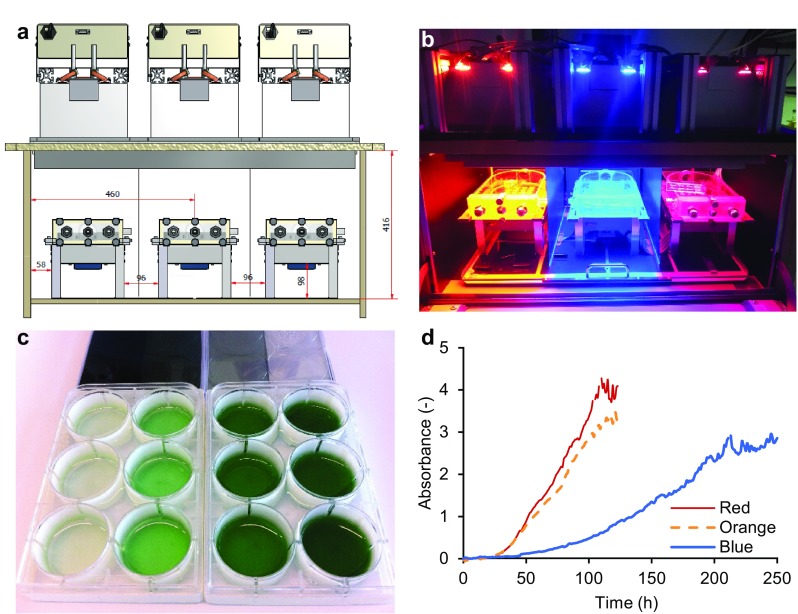
Experimental setup for the batch-culture experiments. **a** Schematic overview of the incubation shaker adapted for controlled experiments with phototrophic microorganisms. LED lights are placed above the incubator, which is partitioned into three color zones each containing a glass vessel mounted on a platform. **b** The incubation shaker in use with orange, blue, and red LED light. **c** Each glass vessel contains two six-well plates with a lid on which neutral density (ND) filters are attached to create different light intensities. Here, we show an example of *Synechocystis* sp. PCC 6803 batch cultures in blue light using 1.2, 0.6, 0.3, and 0.15 ND filters resulting in light intensities of 6, 22, 44, and 70 µmol photons m^−2^ s^−1^, respectively; each light intensity is applied in triplicate. **d** Photodiodes below each well provide automated recording of the light attenuation by each batch culture, from which high-resolution growth curves were calculated (Fig. S2). The growth curves shown here were calculated from the batch cultures in 70 µmol photons m^−2^ s^−1^ of red, orange, and blue light. Each growth curve is the average of three biological replicates

**Fig. 2:**
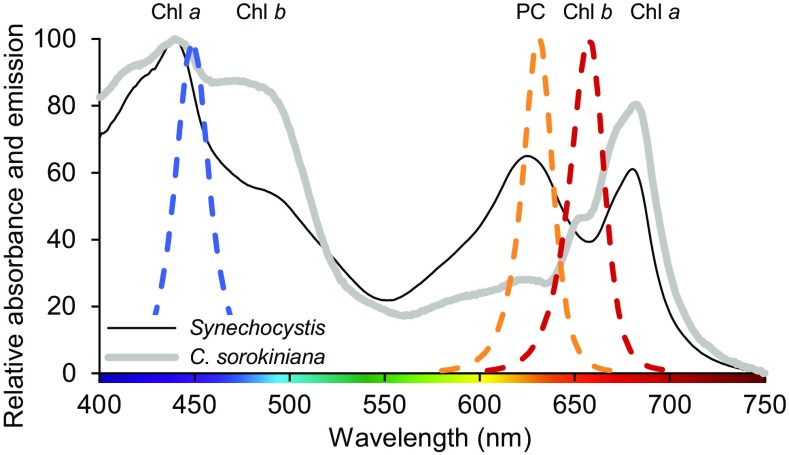
Light absorption spectra of the cyanobacterium *Synechocystis* sp. PCC 6803 (black line) and the green alga *C. sorokiniana* 211-8K (grey line) acclimated to 35 µmol photons m^−2^ s^−1^ white light. Light absorption peaks of Chl *a*, Chl *b*, and phycocyanin (PC) are indicated. Light absorption is normalized to minimum absorbance at 750 nm and maximum absorbance. Dashed lines show emission spectra of the blue (450 nm), orange (625 nm), and red (660 nm) LED light sources used in the experiments, each with a full width at half maximum of ~ 20 nm

To allow semi-high throughput screening of growth rates, an incubation shaker (Multitron Pro, Infors-HT, Velp, The Netherlands) was adapted at the University of Amsterdam Technology Centre for use with LED lighting (Fig. 1). The incubator was divided into three color zones separated by black curtains, with LED lamps (custom made by Philips Lighting B.V., Eindhoven, The Netherlands) positioned above each zone (Fig. 1a, b). The three color zones were illuminated by blue (450 nm), orange (625 nm), or red (660 nm) LED light, each with a full width at half maximum of ~ 20 nm. Emission spectra of the three LED light sources are shown in Fig. 2.

Each color zone contained a glass vessel mounted on a rotating platform. Each glass vessel contained two six-well plates (Corning Costar, Sigma) for batch experiments, with lids on which neutral density (ND) filters (Controllux Lichttechniek BV, Alphen aan den Rijn, The Netherlands) were attached to create different light intensities. The six-well plates were sterilized prior to the experiments using 70% ethanol and air-dried in a laminar flow hood. We applied four light intensities (6, 22, 44, 70 µmol photons m^−2^ s^−1^) per color zone using 1.2, 0.6, 0.3, and 0.15 ND filters, respectively (Fig. 1c). Each light intensity was applied in triplicate. In addition, each glass vessel held a twelve-well plate, without lid, containing sterilized milli-Q water to maintain a high humidity within the vessel. CO_2_-enriched air (2000 ppm) was provided to the incubation shaker through a sterile 0.20 µm Midisart 2000 filter (Sartorius Stedim Biotech GmbH, Göttingen, Germany) to minimize the risk of infection. Temperature in the incubation shaker was maintained at 30 °C and rotation speed of the platform was 100 RPM.

Silicon photodiodes (OSI Optoelectronics, Hawthorne, CA, USA) fixed under each well measured light transmission at hourly intervals, thereby providing automated monitoring of the culture density in the wells. For each photodiode measurement, the applied treatment light was switched off while LED measurement light of 660 nm was switched on at the highest intensity of 185 ± 10 µmol photons m^−2^ s^−1^ for 2–4 s to allow a standardized comparison of the culture density between wells exposed to different treatments.

At the start of the experiments, samples taken from the pre-cultures were diluted in fresh mineral medium to an OD_750_ of 0.04. Subsequently, 5 mL culture of OD_750_ = 0.04 was transferred to each well in the six-well plates to initiate the batch experiments.

### Growth curves

Photodiodes converted the light intensity transmitted through the wells into a voltage signal. To calculate the specific growth rate, first the minimum recording was subtracted to remove the background signal of the photodiodes. The remaining signal can be interpreted as the light intensity *I*(*t*) measured at time *t*, transmitted through a well containing a cell density *X*(*t*) at time *t*. According to Lambert–Beer’s law, this light intensity can be written as,

$$I(t)={I_{\hbox{max} }}\exp \left( { - kX\left( t \right)z} \right),$$

where *I*_max_ is the light intensity transmitted through the well when filled with mineral medium without cells, *k* is the specific light attenuation coefficient of the cells, and *z* is path length. Photodiode reads were normalized by dividing the signal *I*(*t*) by *I*_max_, and then ln-transformed and multiplied by − 1. This gives,

$$\ln \left( {{I_{\hbox{max} }}/I\left( t \right)} \right)=kX(t)z.$$

Hence, in case of exponential cell growth, we obtain the following:

$$\ln \left( {{I_{\hbox{max} }}/I\left( t \right)} \right)=kz{X_0}{e^{\mu t}},$$

where *X*_0_ is the cell density at time 0 and *µ* is the specific growth rate. Accordingly, the transformed signal ln(*I*_max_/*I*(*t*)) was fitted to an exponential trend line, and the specific growth rate, *µ*, was estimated from the coefficient in the exponent. More specifically, we established the exponential growth phase by plotting the transformed signal ln(*I*_max_/*I*(*t*)) on a logarithmic scale and used the data in the exponential growth phase to calculate the specific growth rate. Some illustrations of these calculations for batch experiments at 70 µmol photons m^−2^ s^−1^ of blue and red light are provided in Supplemental Fig. S2.

### Sampling procedures

To assess differences in photophysiology of *Synechocystis* cells exposed to blue, orange, and red light, samples were taken in the exponential growth phase. Each experiment was run two times, using three wells per treatment to obtain triplicate measurements. The first run was used to calculate the specific growth rate and determine the timing of the exponential growth phase based on visual inspection of the transformed signal ln(*I*_max_/*I*(*t*)) plotted on a logarithmic scale. The next run was used to sample the wells during the exponential growth phase.

Samples were taken by opening the culture vessel within the incubator. First, 500 µL was transferred from each well to a fluorescence cuvette prefilled with 2.5 mL 30% glycerol. After mixing by pipetting up and down three times the cuvettes were immediately frozen in liquid nitrogen. This procedure was performed within 20 s to minimize disturbance, ensuring 77 K fluorescence measurements reflected the actual cell status at the moment of sampling. The cuvettes were kept submerged in liquid nitrogen until 77 K fluorescence analysis. Thereafter, an additional 1 mL sample was taken from each well and transferred to a separate cuvette for measurements of absorbance and cell counts.

### Oxygen production rates

O_2_ production by *Synechocystis* cells was measured with samples taken directly from the steady-state continuous culture acclimated to white light. Fresh samples were taken every 30 min and cell densities were determined after each measurement. Chl *a* content was measured spectrophotometrically after extraction in 80% (v/v) acetone/5% (v/v) DMSO (Porra et al. 1989).

Three samples of 3 mL each were transferred simultaneously to three double-walled 3.2 mL glass chambers (UvA TC, Amsterdam) equipped with Firesting Optodes (Pyroscience, Aachen, Germany). Temperature was controlled by continuously pumping 30 °C water through the double wall. LED lamps were positioned on both sides of the three glass chambers to minimize shading. The LED lamps and O_2_-optodes were computer-controlled and the percentage of dissolved O_2_ was converted to µmol O_2_. The value for 0% O_2_ was set by flushing a water sample with pure nitrogen gas for at least 15 min and the 100% saturation value was set by flushing with compressed air (containing 20.9 vol% O_2_) for at least 15 min. O_2_-saturated BG-11 medium of 30 °C contains 234 µM O_2_ at one atmosphere.

Starting with 3 min darkness, samples were exposed to increasing light intensities from 0 to 450 µmol photons m^−2^ s^−1^ while O_2_ concentrations in the chamber were measured. The light intensity was increased every 3 min. The change in O_2_ concentration was calculated 20 s after the light intensity was set until 20 s before the next change in light intensity. O_2_ production rates (*P*) are presented as µmol O_2_ per mg Chl *a* per minute versus light intensity (*I*). The maximum rate of oxygen production (*P*_max_), photosynthetic efficiency (*α*, the initial slope of the curve at low light intensity) and oxygen consumption rate in darkness (*R*_d_) were estimated by fitting a hyperbolic tangent function to the data (Platt and Jassby 1976):

$$P={P_{\hbox{max} ~}}\tanh \left( {\frac{{\alpha ~I}}{{{P_{\hbox{max} }}}}} \right) - {R_{\text{d}}}.$$

The fits were based on a nonlinear least-squares regression using R version 3.3.3 (R Development Core Team 2017).

### Cell counts

Cells were counted and their biovolume measured using a CASY 1 TTC cell counter with a 60 µm capillary (Schärfe Systems GmbH, Reutlingen, Germany), after diluting the samples to ~ 5×10^4^ cells mL^−1^ in Casyton solution.

### Absorption and 77 K fluorescence spectra

Light absorption spectra from 400 to 750 nm were measured with an updated Aminco DW2000 photospectrometer (OLIS, Bogart, GA, USA).

Samples for 77 K fluorescence measurements were analyzed using an OLIS DM45 spectrofluorimeter (OLIS, Bogart, GA, USA) equipped with a dewar cell. Cuvettes were kept submerged in liquid nitrogen and transferred to the measuring cell of the fluorimeter one at a time. We used 440 nm light to excite Chl *a* in the photosystems and 590 nm light to excite PBSs. Fluorescence emission was measured from 630 to 750 nm, yielding emission peaks at 695 and 725 nm for PSII and PSI, respectively, and two merged peaks around 650 to 665 nm for uncoupled PBSs and (allo)phycocyanin not involved in light harvesting. The ratios of the surface areas of these emission peaks are known to reflect the actual protein ratios quite accurately (Murakami 1997; Schuurmans et al. 2017).

## Results

### Light-harvesting pigments

Figure 2 shows light absorption spectra of the cyanobacterium *Synechocystis* sp. PCC 6803 and the green alga *Chlorella sorokiniana* 211-8K, when acclimated to 35 µmol photons m^−2^ s^−1^ of white light. The cyanobacterium has absorption peaks in the blue and red part of the spectrum (440 and 680 nm) due to chlorophyll *a* (Chl *a*), and in the orange part (620 nm) due to phycocyanin. Similar to the cyanobacterium, the green alga also has absorption peaks in the blue and red part of the spectrum (440 and 680 nm) due to Chl *a*. Adjacent to its Chl *a* peaks it has shoulders at 450–500 and 650–670 nm due to Chl *b*, while it lacks pigments specifically absorbing in the orange part of the spectrum. Both species also contain carotenoids, which absorb in the blue-green part (400–525 nm) of the spectrum but are not recognizable as separate peaks.

### Growth rates in blue, orange and red light

To assess the growth rates of *Synechocystis* and *C. sorokiniana* in relation to light absorption by their major light-harvesting pigments, both species were grown in triplicate in batch cultures exposed to four different intensities of either blue (450 nm), orange (625 nm) or red light (660 nm) (Fig. 1).

**Fig. 3:**
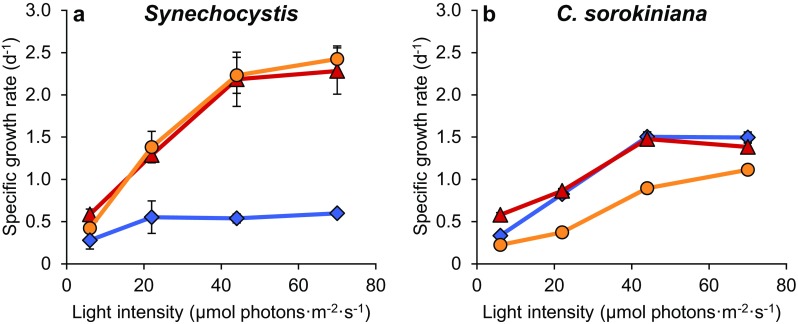
Specific growth rates of the cyanobacterium *Synechocystis* sp. PCC 6803 (**a**) and the green alga *C. sorokiniana* 211-8K (**b**) in batch cultures exposed to blue (blue diamonds), orange (orange circles) and red light (red triangles) at four different light intensities. Results are averages of three biological replicates ± SD; error bars are not visible when SD is smaller than symbol size

Specific growth rates of the cyanobacterium *Synechocystis* sp. PCC 6803 were similar in orange and red light, but much lower in blue light at all four intensities (Fig. 3a). Specific growth rates of the green alga *C. sorokiniana* were similar in blue and red light, but lower in orange light (Fig. 3b).

**Fig. 4:**
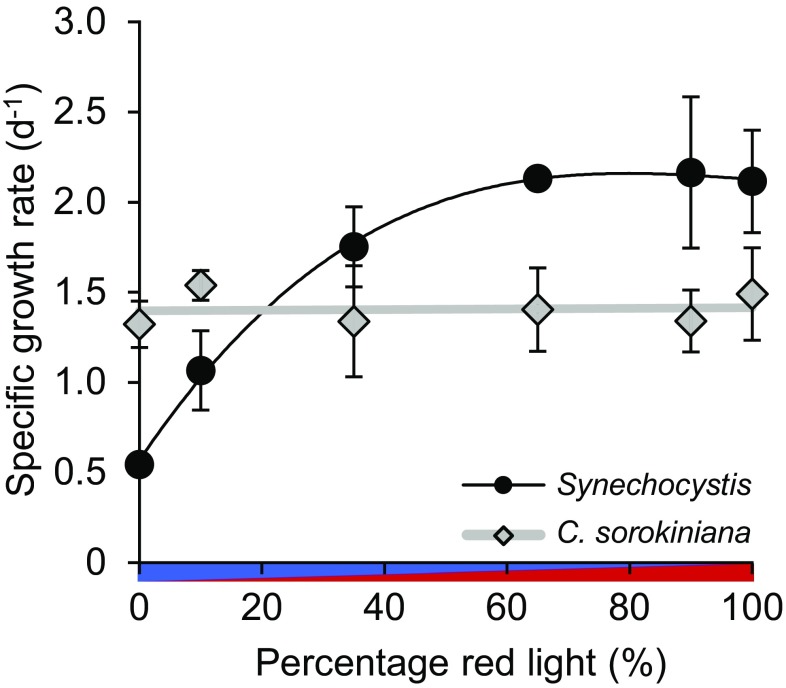
Specific growth rates of the cyanobacterium *Synechocystis* sp. PCC 6803 (black circles) and the green alga *C. sorokiniana* (grey diamonds) in batch cultures exposed to different combinations of red and blue light with a total light intensity of 70 µmol photons m^−2^ s^−1^. Results are averages of three biological replicates ± SD; error bars are not visible when SD is smaller than the symbol size. The data were fitted to a polynomial trendline (using a third-order polynomial for *Synechocystis* and a constant for *C. sorokiniana*)

**Fig. 5:**
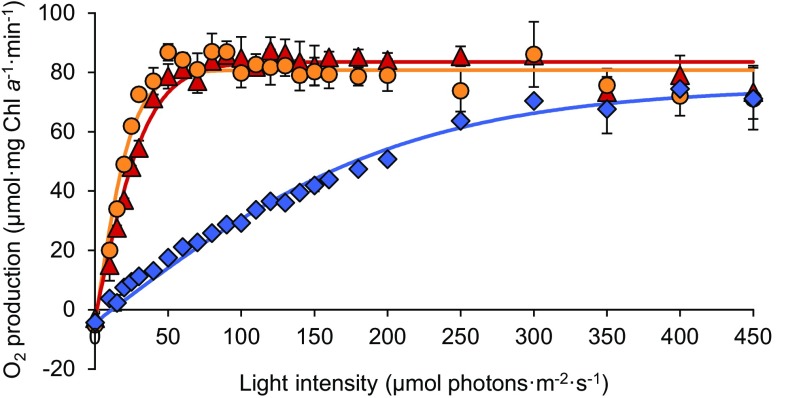
Net O_2_ production by *Synechocystis* sp. PCC 6803 exposed to blue (blue diamonds), orange (orange circles) and red light (red triangles) at different light intensities. *Synechocystis* cells were sampled from a steady-state continuous culture acclimated to white light, and subsequently exposed to the different light colors in an airtight glass chamber to measure O_2_ production using optodes. Data show the averages of three biological replicates ± SD; error bars are not visible when SD is smaller than the symbol size. Solid lines represent fitted *p*–*I* curves using the hyperbolic tangent function; see Table 1 for parameter estimates

Could addition of a minor amount of red light alleviate the inefficiency of blue light in *Synechocystis*? To address this question, we performed additional batch experiments in which *Synechocystis* and *C. sorokiniana* were exposed to mixtures of blue light (450 nm) and red light (660 nm) with a total intensity of 70 µmol photons m^−2^ s^−1^ (Fig. 4). The specific growth rate of *C. sorokiniana* did not change with the percentage red light. In contrast, the specific growth rate of *Synechocystis* strongly increased with the percentage red light, up to 65% red light, and then gradually saturated (Fig. 4). We conjecture that growth rate saturated above 65% red light (i.e., ~ 45 µmol photons m^−2^ s^−1^), because this percentage provided sufficient red light to saturate the photosynthetic rate (see Fig. 5). Hence, we did not observe a distinct enhancement effect in *Synechocystis* when blue and red light were combined, but rather a saturating increase of the growth rate in response to the relative availability of red to blue light.

**Table 1:** Photosynthetic parameters estimated by fitting a hyperbolic tangent to the O_2_ production rates (in µmol O_2_ mg Chl *a*^−1^ min^−1^)

Light source	*P*_max_	α	*R*_d_
Blue	79.5	0.37	4.4
Orange	85.9	3.38	5.1
Red	86.4	2.38	2.9

### Oxygen production is lower in blue light

To investigate the effects of blue light in further detail, O_2_ production rates of *Synechocystis* were measured at increasing intensities of blue (450 nm), orange (625 nm) and red light (660 nm). For this purpose, samples pre-acclimated to white light were transferred to temperature-controlled and airtight flasks, where they were exposed to blue, orange and red light while monitoring O_2_ concentrations.

The O_2_ production rates of *Synechocystis* responded in the same way to light color as the specific growth rates. The increase in O_2_ production with irradiance (i.e., the slope *α* of the *p*–*I* curve) was similar in orange and red light, but much lower in blue light (Fig. 5; Table 1). Photosynthetic O_2_ production saturated at ~ 50 µmol photons m^−2^ s^−1^ in orange and red light but continued to increase with intensity up to ~ 300 µmol photons m^−2^ s^−1^ in blue light. Interestingly, at these high intensities, photosynthetic activity in blue light approached an almost similar maximum O_2_ production rate (i.e., similar *P*_max_) as in orange and red light (Fig. 5; Table 1).

### Absorption spectra and PSI:PSII stoichiometry

**Fig. 6:**
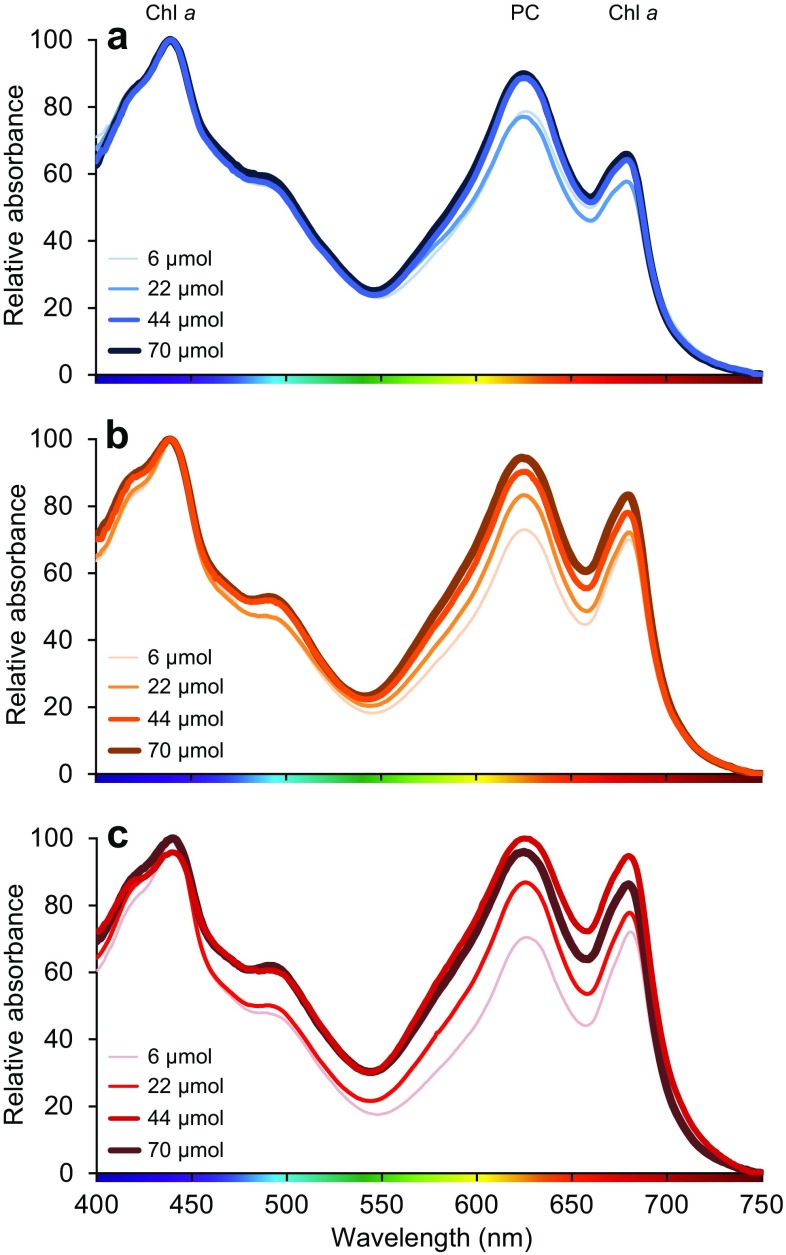
Absorption spectra of *Synechocystis* sp. PCC 6803 in batch cultures exposed to **a** blue light, **b** orange light, and **c** red light at four different light intensities (6, 22, 44 and 70 µmol photons m^−2^ s^−1^). Light absorption peaks by Chl *a* and phycocyanin (PC) are indicated. Spectra show the averages of three biological replicates, and are normalized to minimum absorbance at 750 nm and maximum absorbance at 440 nm

**Fig. 7:**
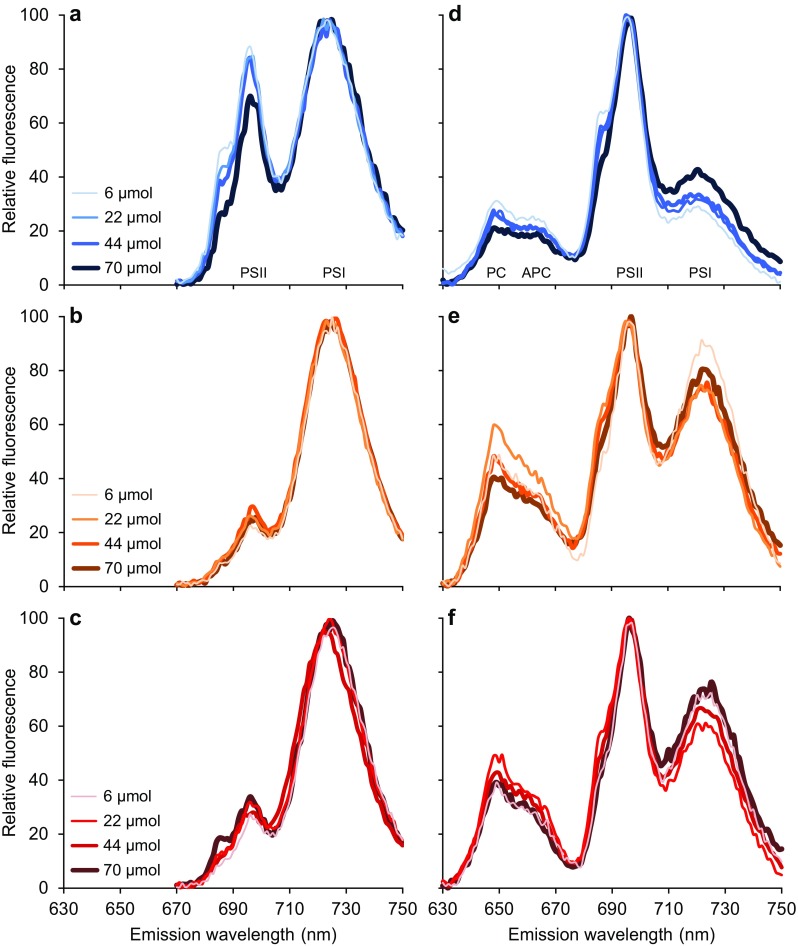
Low-temperature (77 K) fluorescence emission spectra of *Synechocystis* sp. PCC 6803 in batch cultures exposed to **a, d** blue light, **b, e** orange light, and **c, f** red light at four different light intensities (6, 22, 44 and 70 µmol photons m^−2^ s^−1^). **a**–**c** Excitation of Chl *a* at 440 nm yields fluorescence emission peaks at 695 nm for PSII and at 720 nm for PSI. **d**–**f** Excitation of phycocyanin at 590 nm yields fluorescence emission peaks at 695 nm when PBSs are coupled to PSII (state 1) and at 720 nm when coupled to PSI (state 2). PBSs that are decoupled from the photosystems, or phycocyanin that is not incorporated into PBSs, result in fluorescence emission from phycocyanin and allophycocyanin at 650–665 nm. Spectra show the averages of three biological replicates, and are normalized to the minimum and maximum emission of each spectrum

Absorption spectra and 77 K fluorescence emission spectra were measured in batch cultures of *Synechocystis* exposed to either blue, orange or red light at four different light intensities (Figs. 6, 7). The absorption spectra show that the ratio between the phycocyanin peak at 620 nm and Chl *a* peak at 680 nm was higher in blue than in orange and red light, indicating that the phycocyanin content increased relative to Chl *a* when cells were grown in blue light. Furthermore, the absorption shoulder at 470–500 nm, indicative of carotenoids, increased with light intensity in red light and to a lesser extent also in orange light, but not in blue light (Fig. 6).

PSI:PSII ratios of *Synechocystis* were studied by means of low-temperature (77 K) fluorescence spectroscopy (Fig. 7a–c). By freezing cells at 77 K immediately after sampling, the positions of the photosynthetic components are fixed. However, excitation energy can still be transferred to the reaction centers. The subsequent fluorescence emission by the reactions centers of PSI and PSII provides insight into the relative amounts of the two photosystems.

Figure 7a–c show fluorescence emission spectra upon excitation of Chl *a* at 440 nm, which yields fluorescence emission peaks at 695 nm for PSII and at 725 nm for PSI. *Synechocystis* cells acclimated to orange and red light showed very similar results (Fig. 7b, c), with a PSI:PSII fluorescence emission ratio of ~ 4:1 at all four light intensities. In contrast, *Synechocystis* cells acclimated to blue light showed a less characteristic photosystem stoichiometry, with a PSI:PSII fluorescence emission ratio of ~ 1.2:1 at 6, 22 and 44 µmol photons m^−2^ s^−1^ and of ~ 1.4:1 at 70 µmol photons m^−2^ s^−1^, (Fig. 7a). Hence, blue light reduced the PSI:PSII ratio of *Synechocystis*.

### Changes in state transitions

Low-temperature (77 K) fluorescence also provides insight into the coupling of phycobilisomes (PBSs) to the reaction centers of PSI and PSII, and hence into state transitions. Figure 7d–f show fluorescence emission spectra upon excitation at 590 nm of phycocyanin, which is the major phycobiliprotein in PBSs of *Synechocystis* sp. PCC 6803. If PBSs are decoupled from the reaction centers, or phycocyanin is not incorporated into PBSs, excitation of phycocyanin results in fluorescence emission at 650–665 nm. If PBSs are coupled to the reaction centers, the excitation energy is transferred from the PBSs to the reaction centers, resulting in fluorescence emission peaks at 695 nm when coupled to PSII (state 1) or at 725 nm when coupled to PSI (state 2).

In *Synechocystis* cells acclimated to blue light, fluorescence emission by PSII at 695 nm was much higher than fluorescence emission by PSI at 725 nm (Fig. 7d). This indicates that, in blue light, most PBSs transferred their harvested light energy to PSII. By contrast, the difference in fluorescence emission by PSII and PSI was much less pronounced in cells exposed to orange and red light (Fig. 7e, f), indicating that in orange and red light the excitation energy harvested by the PBSs was distributed more or less evenly over both photosystems. We further note that in orange and red light a relatively large fraction of the PBSs seemed not functional in light-harvesting, as fluorescence emission at 650–665 nm was quite prominent (Fig. 7e, f).

### Comparison with other picocyanobacteria

To assess whether the low growth efficiency in blue light is a common phenomenon in cyanobacteria, growth rates were also determined for two marine *Synechococcus* strains, CCY 9201 (formerly known as BS4) and CCY 9202 (formerly BS5). These two picocyanobacteria are of considerable interest, because of their very close genetic relatedness based on the 16S rRNA-ITS operon, while they absorb different parts of the light spectrum because of their different phycobiliproteins (Stomp et al. 2004; Haverkamp et al. 2009). *Synechococcus* sp. CCY 9201 has a similar pigmentation as *Synechocystis* sp. PCC 6803: both utilize the orange-light absorbing phycocyanin in their light-harvesting antennae. In contrast, *Synechococcus* sp. CCY 9202 contains high amounts of phycoerythrin, with which it strongly absorbs in the green part (525–575 nm) of the spectrum (Supplemental Fig. S1).

**Fig. 8:**
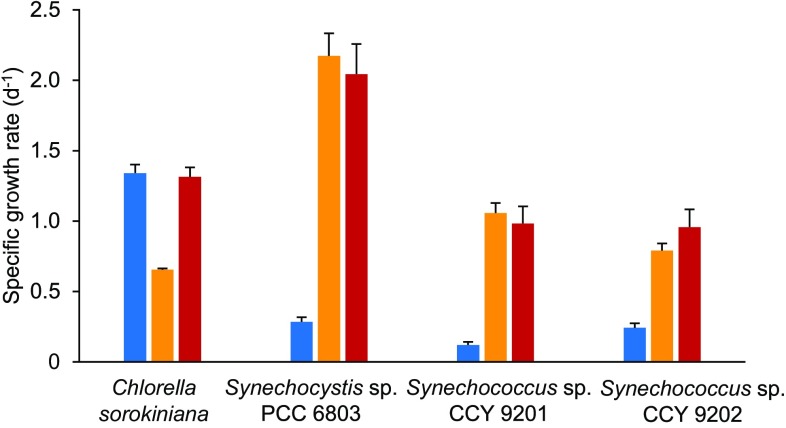
Specific growth rates of the green alga *C. sorokiniana* 211-8K and the cyanobacteria *Synechocystis* sp. PCC 6803, *Synechococcus* sp. CCY 9201 and *Synechococcus* sp. CCY 9202 in batch cultures exposed to blue, orange and red light at an intensity of 44 µmol photons m^−2^ s^−1^. Light absorption spectra of these four species are provided in Supplemental Fig. S1. Results show the averages of three biological replicates ± SD

*Synechococcus* sp. CCY 9201 had its lowest growth rate in blue light, and an eight-fold higher specific growth rate in orange and red light (Fig. 8). The specific growth rate of the phycoerythrin-rich strain CCY 9202 was also lowest in blue light, and it had a three- to four-fold higher specific growth rate in orange and red light, respectively. Hence, both *Synechococcus* strains displayed a qualitatively similar growth response as *Synechocystis* sp. PCC 6803. These results show that the diminished growth rate in blue light is not unique to phycocyanin-rich cyanobacteria, but can also be found in phycoerythrin-rich cyanobacteria.

## Discussion

Our results show that the cyanobacterium *Synechocystis* sp. PCC 6803 absorbs blue light to at least a similar extent as orange and red light (Figs. 2, 6), but uses the absorbed blue light much less effectively for oxygenic photosynthesis and growth (Figs. 3, 4, 5). The poor photosynthetic performance of cyanobacteria in blue light has also been reported by several earlier studies (e.g., Lemasson et al. 1973; Wyman and Fay 1986; Wilde et al. 1997; Tyystjärvi et al. 2002; Wang et al. 2007; Singh et al. 2009; Chen et al. 2010; Choi et al. 2013; Solhaug et al. 2014; Bland and Angenent 2016), but in-depth investigations were still lacking. Our results support the hypothesis that blue light creates an imbalance between the two photosystems, with an excess of energy at the PSI-side and a deficiency at the PSII-side of the photosynthetic electron transport chain of cyanobacteria (e.g., Solhaug et al. 2014; Kirilovsky 2015). This hypothesis is explained below.

In orange and red light, cyanobacteria usually have 2–5 times more PSI than PSII (Fig. 7a–c; see also Singh et al. 2009; Allahverdiyeva et al. 2014; Kirilovsky 2015). Furthermore, cyanobacterial PSI contains around 100 molecules of Chl *a* (Jordan et al. 2001; Kennis et al. 2001), while a single PSII contains only about 35 Chl *a* molecules (Guskov et al. 2009; Umena et al. 2011). Hence, cyanobacteria invest much more of their chlorophyll in PSI than in PSII, and, therefore, PSI will absorb more photons than PSII (e.g., Myers et al. 1980; Fujita 1997). This dissimilarity between the two photosystems is compensated for by the light-harvesting PBSs, which tend to be associated mostly with PSII and hence transfer most of their absorbed photons to PSII (van Thor et al. 1998; Joshua et al. 2005; Mullineaux 2008). In this way, cyanobacteria can maintain linear electron flow by balancing excitation energy between the two photosystems, which will enable the production of both ATP and NADPH required for growth (Allen 2003; Nogales et al. 2012; Mullineaux 2014).

In blue light, the PBSs do not absorb photons very effectively, because the short wavelength of 450 nm does not match the absorption spectrum of phycocyanin (Fig. 2; see also, e.g., Tandeau de Marsac 2003; Six et al. 2007). Hence, in blue light, the PBSs hardly transfer any light energy to PSII. In contrast, the chlorophylls of the more abundant PSI still absorb blue light effectively. Moreover, in cyanobacteria, β-carotenes absorbing blue and green wavelengths are also more abundant in PSI than PSII and further contribute to photosynthetic light harvesting by PSI (Ritz et al. 2000; Takaichi 2011; Stamatakis et al. 2014). Hence, most blue photons are absorbed at PSI, while in comparison PSII has a severe shortage of photons, which will suppress linear electron transport. This is supported by the low O_2_ production rates that we measured when the cyanobacteria were exposed to limiting levels of blue light. Interestingly, once the intensity of blue light was high enough to saturate PSII, the O_2_ production rate in blue light approached the rate found in orange and red light (Fig. 5).

The important role of PBS in the distribution of excitation energy over the two photosystems is illustrated by a study of Campbell (1996) on complementary chromatic adaptation of the cyanobacterium *Calothrix* sp. strain PCC 7601. When acclimated to green light the PBSs of this species contain mostly phycoerythrin, while they contain mostly phycocyanin when adapted to red light. Rates of photosynthesis were similar in both light colors, but 77 K fluorescence emission revealed major differences in the distribution of light by the PBSs. Similar to our experimental results, red light (600–750 nm) applied by Campbell (1996) was absorbed by both Chl *a* and (allo)phycocyanin, and the PBSs of red-light acclimated cells were mostly associated with PSII while Chl *a* harvested light for PSI. In contrast, green light (500–575 nm) was only absorbed by phycoerythrin, and the phycoerythrin-containing PBSs of green-light acclimated cells transferred light energy to both PSI and PSII to maintain the balance between these two photosystems.

Our results indicate that blue-light acclimated cells attempt to restore the balance between the two photosystems in a similar way. Fluorescence emission by the two photosystems after excitation of Chl *a* at 440 nm showed that blue-light acclimated cells decrease their PSI:PSII ratio (Fig. 7a–c), which will increase light absorption by PSII relative to PSI. Furthermore, fluorescence emission after excitation of phycocyanin at 590 nm revealed that blue-light acclimated cells associate even more of their PBSs with PSII than cells acclimated to orange and red light (Fig. 7d–f). All these results indicate that cells exposed to blue light try to increase the transfer of light energy to PSII, to restore the balance. Yet, the low O_2_ production rates indicate that cells in blue light are still unable to sustain a high rate of linear electron flow in comparison to those in orange and red light, unless saturating amounts of blue light are provided.

Previous research has shown that in high CO_2_ conditions, cyanobacteria decrease their PSI:PSII ratio and increase phycocyanin relative to Chl *a* (MacKenzie et al. 2004; Eisenhut et al. 2007). These changes are similar to the changes we observed in blue-light acclimated cultures, indicative of an increased transfer of light energy to PSII at high CO_2_ conditions. Since our experiments were also performed at high CO_2_ conditions, the results of MacKenzie et al. (2004) and Eisenhut et al. (2007) suggest that the photosynthetic efficiency of cyanobacteria in blue light might be even lower under carbon-limiting conditions.

Since red light is also absorbed by Chl *a*, why did red light not give the same results as blue light? The answer is probably related to the wavelength of 660 nm that we used as the red-light source in our experiments. This wavelength is already longer than the 620–650 nm-red light used in many previous studies (Wyman and Fay 1986; Figueroa et al. 1995; Wang et al. 2007; Singh et al. 2009; Chen et al. 2010; Choi et al. 2013; Solhaug et al. 2014; Zavřel et al. 2017). This range of wavelengths around 660 nm is absorbed not only by chlorophyll, but also very effectively by allophycocyanin in the core of the PBS (Lemasson et al. 1973; Glazer and Bryant 1975; MacColl 2004). Hence, the PBSs can still redistribute photons of 660 nm over both photosystems, as indicated by the 77 K fluorescence data of the red-light acclimated cells after excitation of the PBSs (Fig. 7f). One would expect that cells grown with red light at a longer wavelength of 680 nm, which is much less absorbed by allophycocyanin and hence specifically targets Chl *a*, would display similar results as our blue-light exposed cells. Indeed, other studies provide support for this hypothesis. For instance, Murakami (1997) showed that *Synechocystis* sp. PCC 6714 had a substantially lower PSI:PSII ratio when grown at 680-nm red light than at 650-nm red light, resembling the low PSI:PSII ratio that we found for *Synechocystis* sp. PCC 6803 in blue light. Bland and Angenent (2016) found that *Synechocystis* sp. PCC 6803 had a lower specific growth rate at 680 nm than at 660 nm, although the growth rate was even lower in blue light (440 and 460 nm). Blue-light absorbing β-carotenes are more abundant in PSI than in PSII, and in cyanobacteria they contribute to light harvesting only for PSI (Stamatakis et al. 2014). Hence, we speculate that blue light causes an even stronger imbalance between PSI and PSII than red light of 680 nm, which may explain their observation (Bland and Angenent 2016) that the growth rate was even lower in blue light than in red light of 680 nm.

Our findings show that the poor performance in blue light is not specific for the freshwater cyanobacterium *Synechocystis* sp. PCC 6803, but also applies to marine cyanobacteria including both phycocyanin-rich strains such as *Synechococcus* sp. CCY 9201 and phycoerythrin-rich strains such as *Synechococcus* sp. CCY 9202 (Fig. 8). The low rates of growth and photosynthesis in blue light even extend to red algae which also possess PBSs composed of phycocyanin and phycoerythrin (Ley and Butler 1980; Figueroa et al. 1995). Hence, although the chromophores phycoerythrobilin and phycourobilin absorb green light (absorption peak at 545 nm) and blue-green light (495 nm), respectively, these organisms are not able to absorb and redistribute deep blue light (< 450 nm) very effectively between the two photosystems.

In green algae, growth rates in blue and red light are comparable (Fig. 3b; see also Teo et al. 2014; Yan and Zheng 2014; Zhao et al. 2015; de Mooij et al. 2016), thus blue light does not seem to result in an excitation imbalance between their PSII and PSI. Green algae maintain a lower PSI:PSII ratio than cyanobacteria (Murakami et al. 1997; Kirilovsky 2015) and utilize light-harvesting antennae composed of Chl *a* and *b* (Kühlbrandt et al. 1994). Additionally, green algae are able to use blue light more efficiently due to a wider variety of light-harvesting carotenoids, in their photosystems and light-harvesting antennae, which also absorb photons in the blue-green part of the visible light spectrum (Takaichi 2011). Contrary to cyanobacteria, these carotenoids harvest light energy not only for PSI, but also for PSII (Goedheer 1969). Hence, in contrast to the cyanobacterial PBSs, the light-harvesting antennae of green algae absorb both blue and red light, and transfer the absorbed light energy to both PSI and PSII in a balanced way.

Our results help to explain why *Prochlorococcus* species dominate over *Synechococcus* species in the oligotrophic oceans (Partensky et al. 1999; Biller et al. 2015). Because red light is strongly absorbed by water molecules, blue light (400–500 nm) prevails in the deeper water layers of the open ocean (Stomp et al. 2007). Instead of PBSs, *Prochlorococcus* utilizes light-harvesting antennae composed of divinyl Chl *a* and *b* (Chisholm et al. 1992; Ting et al. 2002). Consequently, like green algae, they can balance the amply available blue light between both photosystems. In contrast, *Synechococcus* species utilize blue light much less effectively. The PBSs of marine *Synechococcus* strains of the oligotrophic ocean often contain high contents of phycourobilin (Palenik 2001; Everroad et al. 2006; Six et al. 2007; Grébert et al. 2018), with which they do absorb blue-green light (495 nm). However, none of the phycobiliproteins described so far extends its absorption to the deep-blue wavelengths (< 450 nm) that form a major part of the underwater light spectrum characteristic of the oligotrophic ocean. Hence, not only does blue light offer a suitable habitat for the chlorophyll-based light-harvesting antennae of *Prochlorococcus*, as has been described by many previous studies (Scanlan and West 2002; Ting et al. 2002; Rocap et al. 2003; Stomp et al. 2007), but blue light (< 450 nm) is also less suitable for phycobilisome-containing cyanobacteria such as *Synechococcus*.

## Electronic supplementary material

Below is the link to the electronic supplementary material.

Supplementary material 1. **Fig. S1** Light absorption spectra of three cyanobacteria and a green alga. All four species have absorption peaks for Chl *a* (440 and 680 nm) and carotenoids (400-525 nm). The cyanobacteria *Synechocystis* sp. PCC 6803 (black solid line) and *Synechococcus* sp. CCY 9201 (black dashed line) have an additional absorption peak for phycocyanin (PC) at 620 nm. The cyanobacterium *Synechococcus* sp. CCY 9202 (grey dotted line) has an additional absorption peak for phycoerythrin (PE) at 565 nm. The green alga *C. sorokiniana* (grey solid line) has additional absorption shoulders for Chl *b* at 450-500 nm and 650-670 nm. The species were all grown at 35 μmol photons·m^-2^·s^-1^ of white light (PDF 504 KB)

Supplementary material 2. **Fig. S2** Calculation of the specific growth rate from photodiode reads of batch cultures in the incubation shaker. Here we show examples for three replicate batch cultures of *Synechocystis* sp. PCC 6803 grown in blue (**a**-**d**) and red light (**e**-**h**) at 70 μmol photons·m^-2^·s^-1^. **a,e** The photodiodes record voltage as a measure of light intensity. **b,f** First, the minimum recording was subtracted to remove the background signal of the photodiodes and, subsequently, the photodiode reads were normalized by dividing the data by the recording of mineral medium without cells. **c,g** Next, data were ln-transformed and multiplied by -1. **d,h** Finally, the ln-transformed data were presented on a logarithmic scale and we used the linear part of these growth curves to calculate the specific growth rate by fitting an exponential trendline to the data. The three replicates resulted in three specific growth rates for each experimental condition (PDF 580 KB)

## References

[CR1] Allahverdiyeva Y, Suorsa M, Tikkanen M, Aro EM (2014). Photoprotection of photosystems in fluctuating light intensities. J Exp Bot.

[CR2] Allen JF (2003). Cyclic, pseudocyclic and noncyclic photophosphorylation: new links in the chain. Trends Plant Sci.

[CR3] Arnold W, Oppenheimer JR (1950). Internal conversion in the photosynthetic mechanism of blue-green algae. J Gen Physiol.

[CR4] Biller SJ, Berube PM, Lindell D, Chisholm SW (2015). *Prochlorococcus*: the structure and function of collective diversity. Nature Rev Microbiol.

[CR5] Bland E, Angenent LT (2016). Pigment-targeted light wavelength and intensity promotes efficient photoautotrophic growth of cyanobacteria. Biores Technol.

[CR6] Campbell D (1996). Complementary chromatic adaptation alters photosynthetic strategies in the cyanobacterium *Calothrix*. Microbiology.

[CR7] Campbell D, Hurry V, Clarke AK, Gustafsson P, Öquist G (1998). Chlorophyll fluorescence analysis of cyanobacterial photosynthesis and acclimation. Microbiol Mol Biol Rev.

[CR8] Chen HB, Wu JY, Wang CF, Fu CC, Shieh CJ, Chen CI, Wang CY, Liu YC (2010). Modeling on chlorophyll *a* and phycocyanin production by *Spirulina platensis* under various light-emitting diodes. Biochem Eng J.

[CR9] Chisholm SW, Frankel SL, Goericke R, Olson RJ, Palenik B, Waterbury JB, West-Johnsrud L, Zettler ER (1992). *Prochlorococcus marinus* nov. gen. nov. sp.: an oxyphototrophic marine prokaryote containing divinyl chlorophyll *a* and *b*. Arch Microbiol.

[CR10] Choi CY, Kim NN, Shin HS, Park HG, Cheon S, Kil G (2013). The effect of various wavelengths of light from light-emitting diodes on the antioxidant system of marine cyanobacteria, *Synechococcus* sp. Mol Cell Toxicol.

[CR11] de Mooij T, de Vries G, Latsos C, Wijffels RH, Janssen M (2016). Impact of light color on photobioreactor productivity. Algal Res.

[CR12] Duysens LNM (1951). Transfer of light energy within the pigment systems present in photosynthesizing cells. Nature.

[CR13] Eisenhut M, Von Wobeser EA, Jonas L, Schubert H, Ibelings BW, Bauwe H, Matthijs HCP, Hagemann M (2007). Long-term response toward inorganic carbon limitation in wild type and glycolate turnover mutants of the cyanobacterium *Synechocystis* sp. strain PCC 6803. Plant Physiol.

[CR14] Emerson R, Lewis CM (1942). The photosynthetic efficiency of phycocyanin in *Chroococcus*, and the problem of carotenoid participation in photosynthesis. J Gen Physiol.

[CR15] Engelmann TW (1882). Über sauerstoffausscheidung von pflanzenzellen im mikrospektrum. Pflug Arch Eur J Phys.

[CR16] Engelmann TW (1883). Farbe und assimilation. I. Assimilation findet nur in den farbstoffhaltigen plasmatheilchen statt. II. Näherer zusammenhang zwischen lichtabsorption und assimilation. III. Weitere folgerungen. Bot Zeit.

[CR17] Engelmann TW (1884). Untersuchungen über die quantitativen beziehungen zwischen absorption des lichtes und assimilation in pflanzenzellen. Bot Zeit.

[CR18] Everroad C, Six C, Partensky F, Thomas JC, Holtzendorff J, Wood AM (2006). Biochemical bases of type IV chromatic adaptation in marine *Synechococcus* spp. J Bacteriol.

[CR19] Figueroa FL, Aguilera J, Niell FX (1995). Red and blue light regulation of growth and photosynthetic metabolism in *Porphyra umbilicalis* (Bangiales, Rhodophyta). Eur J Phycol.

[CR20] Fujita Y (1997). A study on the dynamic features of photosystem stoichiometry: accomplishments and problems for future studies. Photosynth Res.

[CR21] Glazer AN, Bryant DA (1975). Allophycocyanin B (λmax 671, 618 nm). Arch Microbiol.

[CR22] Goedheer JC (1969). Energy transfer from carotenoids to chlorophyll in blue-green, red and green algae and greening bean leaves. Biochim Biophys Acta Bioeng.

[CR23] Grébert T, Doré H, Partensky F, Farrant GK, Boss ES, Picheral M, Guidi L, Pesant S, Scanlan DJ, Wincker P, Acinas SG, Kehoe DM, Garczarek L (2018). Light color acclimation is a key process in the global ocean distribution of *Synechococcus* cyanobacteria. Proc Natl Acad Sci USA.

[CR24] Grossman AR, Schaefer MR, Chiang GG, Collier JL (1993). The phycobilisome, a light-harvesting complex responsive to environmental conditions. Microbiol Rev.

[CR25] Guskov A, Kern J, Gabdulkhakov A, Broser M, Zouni A, Saenger W (2009). Cyanobacterial photosystem II at 2.9-Å resolution and the role of quinones, lipids, channels and chloride. Nat Struct Mol Biol.

[CR26] Haverkamp THA, Schouten D, Doeleman M, Wollenzien U, Huisman J, Stal LJ (2009). Colorful microdiversity of *Synechococcus* strains (picocyanobacteria) isolated from the Baltic Sea. ISME J.

[CR27] Huisman J, Matthijs HCP, Visser PM, Balke H, Sigon CAM, Passarge J, Weissing FJ, Mur LR (2002). Principles of the light-limited chemostat: theory and ecological applications. Antonie Leeuwenhoek.

[CR28] Jordan P, Fromme P, Witt HT, Klukas O, Saenger W, Krauß N (2001). Three-dimensional structure of cyanobacterial photosystem I at 2.5 Å resolution. Nature.

[CR29] Jørgensen BB, Cohen Y, Des Marais DJ (1987). Photosynthetic action spectra and adaptation to spectral light distribution in a benthic cyanobacterial mat. Appl Environ Microbiol.

[CR30] Joshua S, Bailey S, Mann NH, Mullineaux CW (2005). Involvement of phycobilisome diffusion in energy quenching in cyanobacteria. Plant Physiol.

[CR31] Kennis JTM, Gobets B, van Stokkum IHM, Dekker JP, van Grondelle R, Fleming GR (2001). Light harvesting by chlorophylls and carotenoids in the photosystem I core complex of *Synechococcus elongatus*: a fluorescence upconversion study. J Phys Chem.

[CR32] Kirilovsky D (2015). Modulating energy arriving at photochemical reaction centers: orange carotenoid protein-related photoprotection and state transitions. Photosynth Res.

[CR33] Kühlbrandt W, Wang DAN, Fujiyoshi Y (1994). Atomic model of plant light-harvesting complex by electron crystallography. Nature.

[CR34] Lemasson C, Tandeau de Marsac N, Cohen-Bazire G (1973). Role of allophycocyanin as light-harvesting pigment in cyanobacteria. Proc Natl Acad Sci USA.

[CR35] Ley AC, Butler WL (1980). Effects of chromatic adaptation on the photochemical apparatus of photosynthesis in *Porphyridium cruentum*. Plant Physiol.

[CR36] MacColl R (2004). Allophycocyanin and energy transfer. Biochim Biophys Acta Bioeng.

[CR37] MacKenzie TDB, Burns RA, Campbell DA (2004). Carbon status constrains light acclimation in the cyanobacterium *Synechococcus elongatus*. Plant Physiol.

[CR38] Mullineaux CW (2008). Phycobilisome-reaction centre interaction in cyanobacteria. Photosynth Res.

[CR39] Mullineaux CW (2014). Co-existence of photosynthetic and respiratory activities in cyanobacterial thylakoid membranes. Biochim Biophys Acta.

[CR40] Murakami A (1997). Quantitative analysis of 77 K fluorescence emission spectra in *Synechocystis* sp. PCC 6714 and *Chlamydomonas reinhardtii* with variable PS I/PS II stoichiometries. Photosynth Res.

[CR41] Murakami A, Kim SJ, Fujita Y (1997). Changes in photosystem stoichiometry in response to environmental conditions for cell growth observed with the cyanophyte *Synechocystis* PCC 6714. Plant Cell Physiol.

[CR42] Myers J, Graham JR, Wang RT (1980). Light harvesting in *Anacystis nidulans* studied in pigment mutants. Plant Physiol.

[CR43] Nogales J, Gudmundsson S, Knight EM, Palsson BO, Thiele I (2012). Detailing the optimality of photosynthesis in cyanobacteria through systems biology analysis. Proc Natl Acad Sci USA.

[CR44] Ogawa T, Misumi M, Sonoike K (2017). Estimation of photosynthesis in cyanobacteria by pulse-amplitude modulation chlorophyll fluorescence: problems and solutions. Photosynth Res.

[CR45] Palenik B (2001). Chromatic adaptation in marine *Synechococcus* strains. Appl Environ Microbiol.

[CR46] Partensky F, Hess WR, Vaulot D (1999). *Prochlorococcus*, a marine photosynthetic prokaryote of global significance. Microbiol Mol Biol Rev.

[CR47] Platt T, Jassby AD (1976). The relationship between photosynthesis and light for natural assemblages of coastal marine phytoplankton. J Phycol.

[CR48] Porra RJ, Thompson WA, Kriedemann PE (1989). Determination of accurate extinction coefficients and simultaneous equations for assaying chlorophylls a and b extracted with four different solvents: verification of the concentration of chlorophyll standards by atomic absorption spectroscopy. Biochim Biophys Acta Bioeng.

[CR49] Pulich WM, van Baalen C (1974). Growth requirements of blue-green algae under blue light conditions. Arch Microbiol.

[CR50] R Development Core Team (2017). R: a language and environment for statistical computing.

[CR51] Ritz T, Damjanović A, Schulten K, Zhang JP, Koyama Y (2000). Efficient light harvesting through carotenoids. Photosynth Res.

[CR52] Rocap G, Larimer FW, Lamerdin J, Malfatti S, Chain P, Ahlgren NA, Arellano A, Coleman M, Hauser L, Hess WR (2003). Genome divergence in two *Prochlorococcus* ecotypes reflects oceanic niche differentiation. Nature.

[CR53] Scanlan DJ, West NJ (2002). Molecular ecology of the marine cyanobacterial genera *Prochlorococcus* and *Synechococcus*. FEMS Microbiol Ecol.

[CR54] Schuurmans RM, Matthijs HCP, Hellingwerf KJ (2017). Transition from exponential to linear photoautotrophic growth changes the physiology of *Synechocystis* sp. PCC 6803. Photosynth Res.

[CR55] Shen G, Boussiba S, Vermaas WF (1993). *Synechocystis* sp. PCC 6803 strains lacking photosystem I and phycobilisome function. Plant Cell.

[CR56] Singh AK, Bhattacharyya-Pakrasi M, Elvitigala T, Ghosh B, Aurora R, Pakrasi HB (2009). A systems-level analysis of the effects of light quality on the metabolism of a cyanobacterium. Plant Physiol.

[CR57] Six C, Thomas JC, Garczarek L, Ostrowski M, Dufresne A, Blot N, Scanlan DJ, Partensky F (2007). Diversity and evolution of phycobilisomes in marine *Synechococcus* spp.: a comparative genomics study. Genome Biol.

[CR58] Solhaug KA, Xie L, Gauslaa Y (2014). Unequal allocation of excitation energy between photosystem II and I reduces cyanolichen photosynthesis in blue light. Plant Cell Physiol.

[CR59] Stadnichuk IN, Tropin IV (2017). Phycobiliproteins: structure, functions and biotechnological applications. Appl Biochem Microbiol.

[CR60] Stamatakis K, Tsimilli-Michael M, Papageorgiou GC (2014). On the question of the light-harvesting role of β-carotene in photosystem II and photosystem I core complexes. Plant Physiol Biochem.

[CR61] Stomp M, Huisman J, De Jongh F, Veraart AJ, Gerla D, Rijkeboer M, Ibelings BW, Wollenzien UI, Stal LJ (2004). Adaptive divergence in pigment composition promotes phytoplankton biodiversity. Nature.

[CR62] Stomp M, Huisman J, Stal LJ, Matthijs HCP (2007). Colorful niches of phototrophic microorganisms shaped by vibrations of the water molecule. ISME J.

[CR63] Takaichi S (2011). Carotenoids in algae: distributions, biosyntheses and functions. Mar Drugs.

[CR64] Tamary E, Kiss V, Nevo R, Adam Z, Bernát G, Rexroth S, Rögner M, Reich Z (2012). Structural and functional alterations of cyanobacterial phycobilisomes induced by high-light stress. Biochim Biophys Acta.

[CR65] Tandeau de Marsac N (2003). Phycobiliproteins and phycobilisomes: the early observations. Photosynth Res.

[CR66] Teo CL, Atta M, Bukhari A, Taisir M, Yusuf AM, Idris A (2014). Enhancing growth and lipid production of marine microalgae for biodiesel production via the use of different LED wavelengths. Biores Tech.

[CR67] Ting CS, Rocap G, King J, Chisholm SW (2002). Cyanobacterial photosynthesis in the oceans: the origins and significance of divergent light-harvesting strategies. Trends Microbiol.

[CR68] Tyystjärvi T, Tuominen I, Herranen M, Aro EM, Tyystjärvi E (2002). Action spectrum of *psbA* gene transcription is similar to that of photoinhibition in *Synechocystis* sp. PCC 6803. FEBS Lett.

[CR69] Umena Y, Kawakami K, Shen J, Kamiya N (2011). Crystal structure of oxygen-evolving photosystem II at a resolution of 1.9 Å. Nature.

[CR70] van Thor JJ, Mullineaux CW, Matthijs HCP, Hellingwerf KJ (1998). Light harvesting and state transitions in cyanobacteria. Plant Biol.

[CR71] Wang CY, Fu CC, Liu YC (2007). Effects of using light-emitting diodes on the cultivation of *Spirulina platensis*. Biochem Eng J.

[CR72] Watanabe M, Ikeuchi M (2013). Phycobilisome: architecture of a light-harvesting supercomplex. Photosynth Res.

[CR73] Wilde A, Churin Y, Schubert H, Börner T (1997). Disruption of a *Synechocystis* sp. PCC 6803 gene with partial similarity to phytochrome genes alters growth under changing light qualities. FEBS Lett.

[CR74] Wyman M, Fay P (1986). Underwater light climate and the growth and pigmentation of planktonic blue-green algae (Cyanobacteria) II. The influence of light quality. Proc R Soc Lond Ser B.

[CR75] Yan C, Zheng Z (2014). Performance of mixed LED light wavelengths on biogas upgrade and biogas fluid removal by microalga *Chlorella* sp. Appl Energy.

[CR76] Zavřel T, Očenášová P, Červený J (2017). Phenotypic characterization of *Synechocystis* sp. PCC 6803 substrains reveals differences in sensitivity to abiotic stress. PLoS ONE.

[CR77] Zhao Y, Sun S, Hu C, Zhang H, Xu J, Ping L (2015). Performance of three microalgal strains in biogas slurry purification and biogas upgrade in response to various mixed light-emitting diode light wavelengths. Biores Tech.

