# Supplementary figures and images for: Blue light reduces photosynthetic efficiency of cyanobacteria through an imbalance between photosystems I and II
Source: Photosynth Res. 2018 Jul 19;138(2):177–89. doi: 10.1007/s11120-018-0561-5 (PMC6208612; doi:10.1007/s11120-018-0561-5)

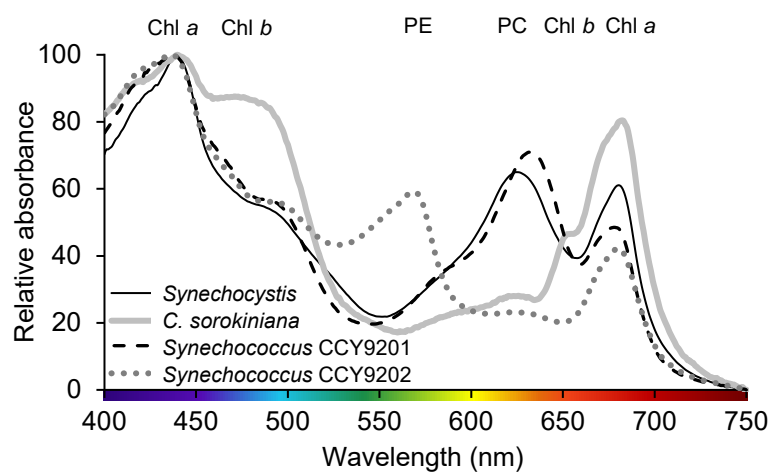

Supplement: Supplementary file 1 — Supplementary material 1. Fig. S1 Light absorption spectra of three cyanobacteria and a green alga. All four species have absorption peaks for Chl a (440 and 680 nm) and carotenoids (400-525 nm). The cyanobacteria Synechocystis sp. PCC 6803 (black solid line) and Synechococcus sp. CCY 9201 (black dashed line) have an additional absorption peak for phycocyanin (PC) at 620 nm. The cyanobacterium Synechococcus sp. CCY 9202 (grey dotted line) has an additional absorption peak for phycoerythrin (PE) at 565 nm. The green alga C. sorokiniana (grey solid line) has additional absorption shoulders for Chl b at 450-500 nm and 650-670 nm. The species were all grown at 35 μmol photons·m-2·s-1 of white light (PDF 504 KB) [file 11120_2018_561_MOESM1_ESM.pdf]

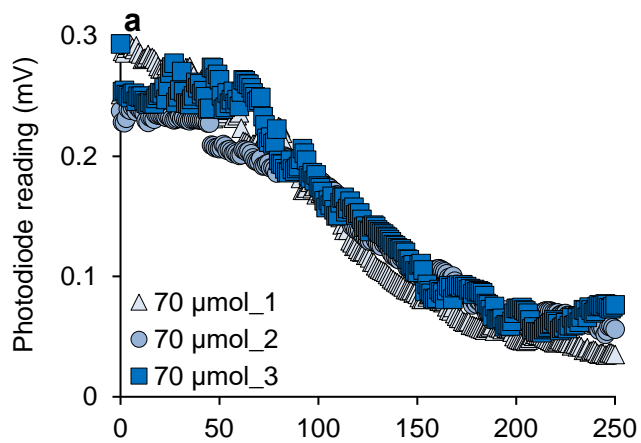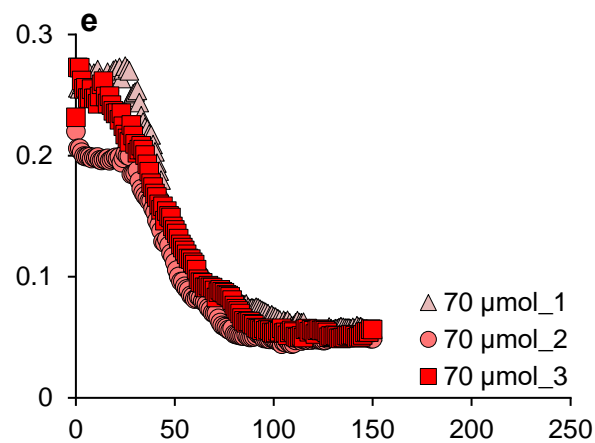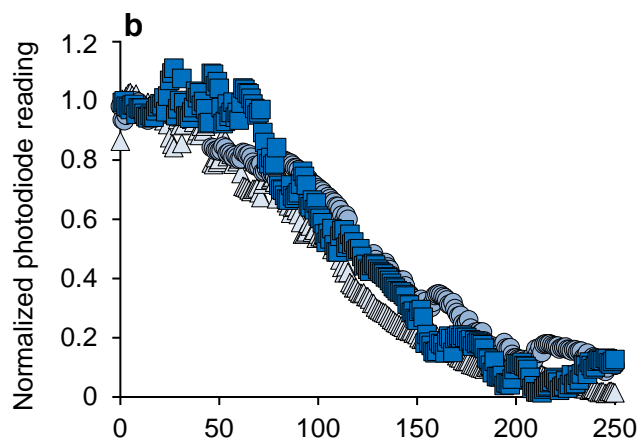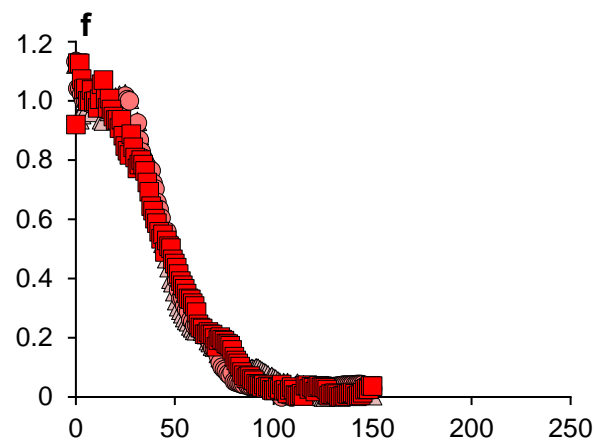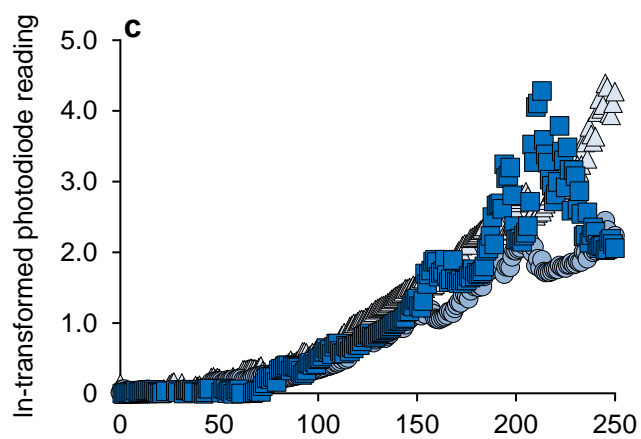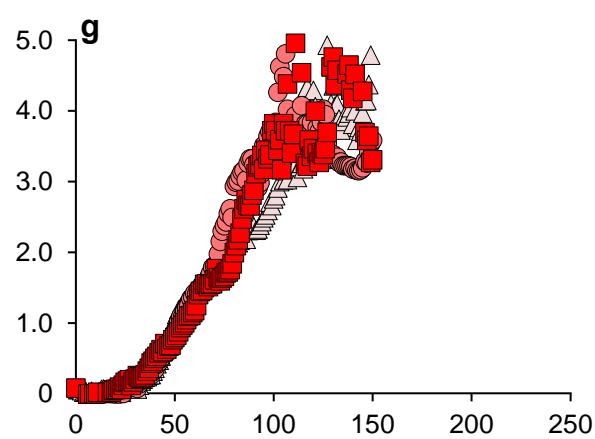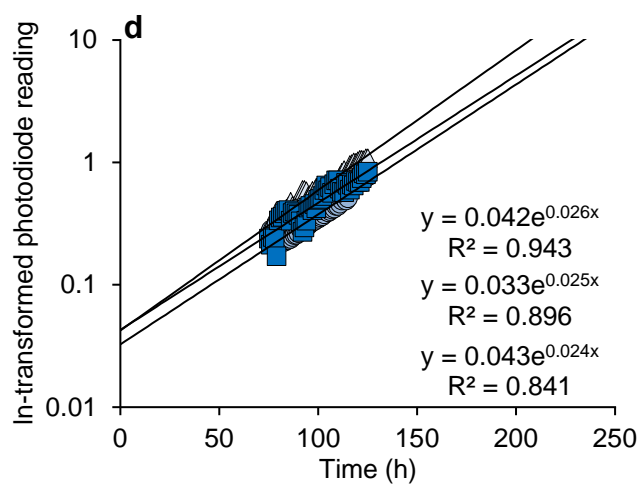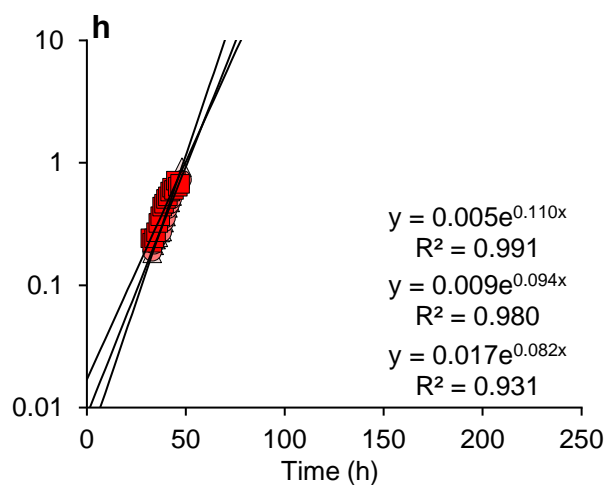

Supplement: Supplementary file 2 — Supplementary material 2. Fig. S2 Calculation of the specific growth rate from photodiode reads of batch cultures in the incubation shaker. Here we show examples for three replicate batch cultures of Synechocystis sp. PCC 6803 grown in blue (a-d) and red light (e-h) at 70 μmol photons·m-2·s-1. a,e The photodiodes record voltage as a measure of light intensity. b,f First, the minimum recording was subtracted to remove the background signal of the photodiodes and, subsequently, the photodiode reads were normalized by dividing the data by the recording of mineral medium without cells. c,g Next, data were ln-transformed and multiplied by -1. d,h Finally, the ln-transformed data were presented on a logarithmic scale and we used the linear part of these growth curves to calculate the specific growth rate by fitting an exponential trendline to the data. The three replicates resulted in three specific growth rates for each experimental condition (PDF 580 KB) [file 11120_2018_561_MOESM2_ESM.pdf]
